# Characterization of the Oral and Esophageal Microbiota in Esophageal Precancerous Lesions and Squamous Cell Carcinoma

**DOI:** 10.3389/fcimb.2021.714162

**Published:** 2021-09-15

**Authors:** Zhengqi Li, Lizhou Dou, Yueming Zhang, Shun He, Deli Zhao, Changqing Hao, Guohui Song, Wei Zhang, Yong Liu, Guiqi Wang

**Affiliations:** ^1^Department of Endoscopy, National Cancer Center/National Clinical Research Center for Cancer/Cancer Hospital, Chinese Academy of Medical Sciences and Peking Union Medical College, Beijing, China; ^2^Cancer Center, Feicheng People’s Hospital, Feicheng, China; ^3^Department of Endoscopy, Linzhou Cancer Hospital, Linzhou, China; ^4^Department of Epidemiology, Cancer Institute/Hospital of Ci County, Handan, China; ^5^Department of Endoscopy, National Cancer Center/National Clinical Research Center for Cancer/Cancer Hospital & Shenzhen Hospital, Chinese Academy of Medical Sciences and Peking Union Medical College, Shenzhen, China

**Keywords:** characterization, oral and esophageal, microbiota, esophageal precancerous lesions, esophageal squamous cell carcinoma

## Abstract

Important evidence indicates that the microbiota plays a key role in esophageal squamous cell carcinoma (ESCC). Here, paired saliva and brush specimens were obtained from 276 participants undergoing upper gastrointestinal endoscopic examination before or during screening for upper gastrointestinal (UGI) cancer. The esophageal microbiota was investigated by 16S rRNA gene profiling and next-generation sequencing. We observed that as the disease progressed, the α diversity in the saliva and cell brush samples decreased. Linear discriminant analysis effect size (LEfSe) results showed that in both the saliva and cell brush specimens, *Granulicatella, Rothia, Streptococcus*, *Gemella, Leptotrichia* and *Schaalia* were common biomarkers in patients with low-grade dysplasia, *Lactobacillus* was a common biomarker in patients with high-grade dysplasia, and *Bosea*, *Solobacterium, Gemella*, and *Peptostreptococcus* were common biomarkers in patients with esophageal cancer. The top 3 genera in the saliva and cell brush specimens had areas under the curve (AUCs) of 87.16 and 89.13%, respectively, to distinguish ESCC patients from normal people. The PICRUSt2 results identified in brush samples that patients with ESCC had decreased nitrate reductase functions. Our results suggest that future studies can focus on the function of the characteristic bacteria in ESCC.

## Introduction

Esophageal cancer is the sixth deadliest cancer in the world. Ninety percent of the 456,000 esophageal cancer cases reported each year (Abnet et al., 2018) are caused by esophageal squamous cell neoplasia (ESCN). China accounts for approximately half of all esophageal squamous cell cancer (ESCC) cases worldwide (Liu et al., 2019) due to its high incidence rate and China’s large population. Early diagnosis is key to improving the prognosis of esophageal cancer (Codipilly et al., 2018). However, early ESCN is often difficult to detect, with the gold standard for the diagnosis of early ESCN being pathological biopsy obtained by gastroendoscopy (Di Pietro et al., 2018). Although China has made improvements in esophageal cancer screening in recent years (Chen et al., 2020), endoscopic examination is relatively expensive, painful and invasive, and not everyone accepts screening. Therefore, it is necessary to develop a new noninvasive screening tool to detect ESCC.

The occurrence of ESCC is believed to be due to multiple factors (Abnet et al., 2018). The microbiome exerts significant effects on human health and disease (Goodrich et al., 2017; Panduro et al., 2017). In humans, the intestines, skin, respiratory tract, reproductive tract and other body parts in contact with the environment are naturally colonized by microbial flora. With the increase in microbiological research and the growth of high-throughput sequencing technology in recent years, the identification of digestive tract flora has been greatly improved (Ju and Zhang, 2015; Bhatt et al., 2017). Previous studies have confirmed that changes in the composition of the local microbiota in the digestive tract may be a risk element for esophageal cancer (Meng et al., 2018). However, because of the difficulties in obtaining samples from this region of the body compared to other parts of the body (such as the stomach and intestines), the number of studies on the esophageal microbiota is limited. In addition, because of differences in the incidence rates of esophageal cancer between Western and Eastern countries, the ESCC microbiota has been studied less frequently than the esophageal adenocarcinoma microbiota (Di Pilato et al., 2016; Zhang and Pan, 2020), with few studies having investigated the oral and esophageal microbiota in the progression of ESCC (Chen et al., 2015).

The importance of investigating the microbiota in the oral cavity and on the surface of precancerous lesions in the esophagus is based on two major considerations. First, published studies on precancerous lesions of ESCC are insufficient, and the sample sizes used were small (Li et al., 2020). Thus, a study with a large sample size is needed to explore changes in the microbiota with regard to the development of ESCC. Second, although studies have shown that the oral microbiota in ESCC patients is different from that observed in normal populations (Peters et al., 2017; Wang et al., 2019; Liu et al., 2020; Zhao et al., 2020), the characteristics of the oral microbiota in precancerous lesions of ESCC and the difference between matched samples of the oral microbiota and esophageal microbiota in precancerous lesions of ESCC have not been reported. Thus, the goal of the present study was to address the above two challenges.

## Materials and Methods

### Study Participants

The present study was based on endoscopic screening conducted in the Chinese Upper Gastrointestinal (UGI) Cancer Project. We prospectively recruited 277 participants aged 43–81 years who had a pathologic diagnosis of normal, low-grade dysplasia (LGD), high-grade dysplasia (HGD) or ESCC at the Cancer Hospital of the Chinese Academy of Medical Sciences (Beijing, China), Linzhou Cancer Hospital in Henan Province, China, the Cancer Institute/Hospital of Ci County in Hebei Province and Feicheng People’s Hospital in Shandong Province, China, from July 2018 to September 2020. All pathological diagnoses were made by pathologists based on the 2019 WHO classification of tumors of the digestive system (Nagtegaal et al., 2019). Notably, we only sampled lesions in the thoracic esophagus, which is approximately 20–38 cm from the central incisor. All participants were located in Northern China. All participants in this study were provided clear notice and signed written informed consent. The Clinical Trials Center of the National Cancer Center approved and oversaw this research (NO. 19/191–1975). Participants’ sex, age, smoking and alcohol drinking status, history of digestive system disease medication history in the previous month and oral condition were collected by experienced staff.

Patients who had other systemic diseases; were taking antibiotics, proton pump inhibitors, prebiotics or other preparations in the previous month; or had a history of oral ulcers in the previous month were excluded from the study.

### Sample Collection

Paired saliva and brush specimens were obtained from 277 participants undergoing upper gastroenterology endoscopic examination before and during screening for UGI cancer.

Before endoscopy for UGI cancer screening, the patients were told not to eat food, drink liquids, or brush their teeth. A questionnaire was administered by staff, and a 5-mL saliva sample was collected from the eligible patient in a saliva collection tube (SAL2000 L, Zeesan, Xiamen, China).

Brush specimens were collected using sterile brushes. In normal patients, a brush sample was obtained at a location 25 cm from the central incisor, with care taken to let the brush fully contact the four walls of the esophagus. For patients with precancerous lesions or ESCC, the brush contacted only the lesion. After a brush sample was collected, the brush head was removed and placed into a sterile tube (Cryovial, 3.0-mL cryogenic tube). All paired specimens were stored at -80°C and later transported to the laboratory on dry ice.

### DNA Extraction, Amplification, and Sequencing

Total genomic DNA was extracted from saliva and esophageal brush specimens using the CTAB method (Zhang and Wang, 2017). DNA concentration and purity were assessed on 1% agarose gels, and based on the observed concentration, the DNA samples were diluted to 1 ng/µL using sterile water. The V4 region of the 16S ribosomal RNA (rRNA) gene was polymerase chain reaction (PCR) amplified using bacterial primers (5’-GTGCCAGCMGCCGCGGTAA-3’) and reverse primers (5’-GGACTACHVGGGTWTCTAAT-3’) with incorporated barcodes. All PCRs were carried out in 30 µL reactions with 15 µL of Phusion^®^ High-Fidelity PCR master mix (New England Biolabs), 0.2 µM each of the forward and reverse primers, and approximately 10 ng of template DNA.

Thermal cycling consisted of initial denaturation at 98°C for 1 min followed by 30 cycles of denaturation at 98°C for 10 s, annealing at 50°C for 30 s, and elongation at 72°C for 30 s, with a final extension performed at 72°C for 5 min. Then, an equal volume of 1× loading buffer (containing SYBR green) was mixed with the PCR products, and electrophoresis was performed on a 2% agarose gel for amplicon detection. Subsequently, the PCR products were mixed in equidensity ratios and then purified with a GeneJET™ gel extraction kit (Thermo Scientific). Sequencing libraries were generated using an Ion Plus Fragment Library Kit 48 rxns (Thermo Scientific) following the manufacturer’s recommendations. The library quality was assessed with a Qubit 2.0 Fluorometer (Thermo Scientific) and then sequenced on an Ion S5TM XL platform to generate 600 bp single-end reads.

### Sequence Processing and Taxonomic Classification

In total, 553 of the 554 collected specimens were successfully amplified (one brush specimen in the ESCC group failed to amplify). To ensure that every specimen remained paired, the saliva specimen paired with the brush specimen that failed to amplify in the ESCC group was excluded from analysis. The remaining 552 specimens were processed using the Quantitative Insights into Microbial Ecology (QIIME2, https://qiime2.org/) platform. Raw sequences were subjected to strict quality control and feature table construction using Cutadapt (Martin, 2011) (V1.9.1, http://cutadapt.readthedocs.io/en/stable/). The sequencing method was single-ended sequencing. The maximum length that the machine could read was 600 bp. Reads were analyzed using Vsearch (version 2.17.1) and Usearch (version 11) (Rognes et al., 2016; Edgar, 2018). After quality filtering and chimera removal, clean sequences and exact amplicon sequence variants (ASVs) were resolved using Unoise3. Taxonomy was assigned to ASVs using Blast+-blastn with -e 0.00001 with the eHOMD database (http://www.homd.org). Python’s scikit-bio module was used to calculate the Shannon index and Chao1 index. Then, linear discriminant analysis effect size (LEfSe) was performed to find biomarker(s) differentially represented between different groups (Segata et al., 2011). Picrust2 was used to predict the function of the representative sequence (Douglas et al., 2020) and extract the K number related to the nitrite metabolism process.

### Statistical Analysis

We compared demographic and other characteristics between normal, LGD, HGD and ESCC patients with t-tests and chi-squared tests. The program R Studio (Version 4.1.0; R Foundation for Statistical Computing, Vienna, Austria) was used to perform all statistical analyses. The LSD test function of the R software agricolae package was used to analyze the difference in the α diversity index of ASV, and the fdr method was used to correct the P value (P=0.05). The vegdist vegan package was used to calculate the Bray-Curtis distance matrix at the genus level. Then, the cmdscale function was used for principal coordinate analysis, and perMANOVA was conducted by the adonis function. LEfSe was performed to identify microbes associated with tumor status. Microbiota with a linear discriminant analysis (LDA) score greater than 3.0 were defined as different genera. The randomForest function was used to perform random forest analysis. Extract the features in the random forest analysis results. Sort the features in descending order according to the MeanDecreaseAccuracy value. Take the top 3, 5, 10, 20, and 30 features in the ranking results to perform random forest analysis. Then, the pROC package was used to perform ROC analysis based on the random forest results, and ggroc was used to draw the result graph. According to the results of random forest and ROC, the species was selected with features of 30 to plot. The length of the bar graph was the value of MeanDecreaseAccuracy in the random forest analysis. Then, the abundance of the species corresponding to features in each sample group was counted. The calculation method for the difference in nitrite metabolism was the same as that for the difference in the α diversity index. DESeq2 was used to analyze differences between species at the genus level with the same symptoms in saliva and brush samples. The R packages used in the above data processing process were data.table, reshape2, stringr, and aplot. Unless otherwise specified, all figures in the article were drawn using the ggplot2 package in R software.

## Results

### Baseline Characteristics of the Participants

The 276 patients included in the statistical analysis were divided into 4 groups according to pathology: the normal group (82 patients), the LGD group (60 patients), the HGD group (64 patients) and the SCC group (70 patients). No significant differences in age or BMI were observed among the groups. In addition, no significant differences were detected for the baseline characteristics of alcohol consumption and smoking status (**Table 1**).

**Table 1:** The baseline characteristics of participants.

Variables	Normal (n = 82)	LGD (n = 60)	HGD (n = 64)	ESCC (n = 70)	P
Age, years (x̅ ± s)	58.51 ± 5.82	62.40 ± 6.45	62.47 ± 6.60	63.46 ± 6.77	0.331
Sex, n (%)					0.504
Male	48 (58.5)	34 (56.7)	34 (53.1)	46 (65.7)	
Female	34 (41.5)	26 (43.3)	30 (46.9)	24 (34.3)	
BMI (x̅ ± s)	24.32 ± 3.12	23.58 ± 2.68	23.15 ± 3.26	23.56 ± 2.55	0.106
Current smoking, n (%)	18 (22.0)	12 (20.0)	16 (25.0)	24 (34.3)	0.225
Current alcohol consumption, n (%)	16 (19.5)	19 (31.7)	15 (23.4)	26 (37.1)	0.074

### Microbial Differences Among the Saliva Specimens From the Different Pathological Groups

The saliva specimens from the different pathological groups (the normal, LGD, HGD, and SCC groups) are indicated as NS, LGDS, HGDS and SCCS, respectively.

**Figure 1:**
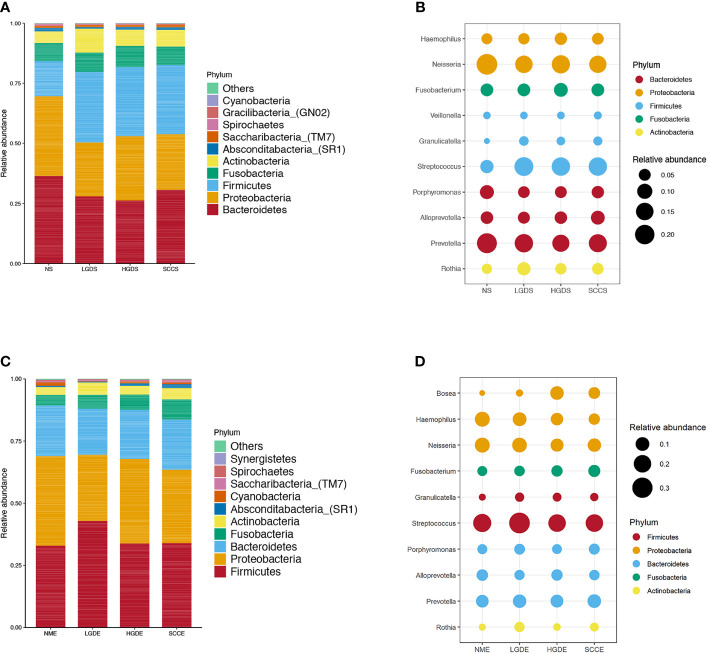
The microbiota relative abundances of all groups. **(A)** The relative abundances of phyla in the NS, LGDS, HGDS and SCCS groups. **(B)** The relative abundances of genera in the NS, LGDS, HGDS and SCCS groups. **(C)** The relative abundances of phyla in the NME, LGDE, HGDE and SCCE groups. **(D)** The relative abundances of genera in the NME, LGDE, HGDE and SCCE groups.

**Figure 2:**
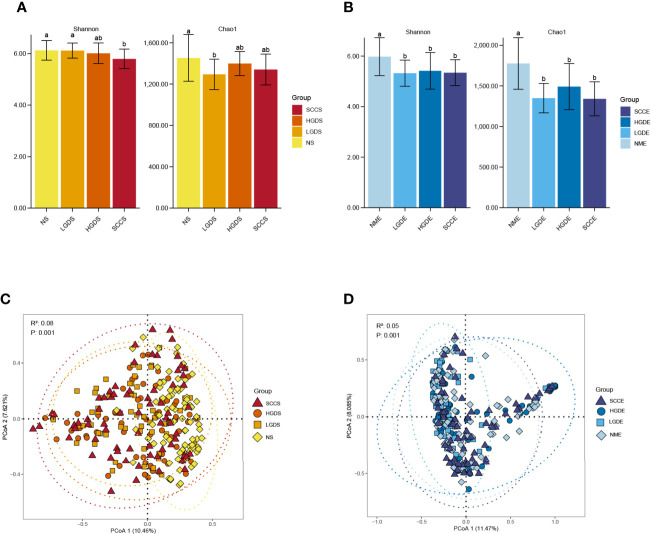
The α diversities and β diversities of microbiota in all groups. The LSD test function of the R software agricolae package was used to analyze the difference in the α diversity index of ASV, and the fdr method was used to correct the P value (P=0.05). The vegdist vegan package was used to calculate the Bray-Curtis distance matrix at the genus level. Then, the cmdscale function was used for principal coordinate analysis, and the perMANOVA test was conducted by the adonis function. **(A)** The Shannon and Chao1 indices for the saliva samples. **(B)** The Shannon and Chao1 indices for the brush samples. **(C)** The Bray-Curtis distance matrix at the genus level for the saliva samples. **(D)** The Bray-Curtis distance matrix at the genus level for the brush samples. Different small letters in the bar chart represent statistical differences between the two sets of data.

**Figures 1A, B** show the compositions of the microbiota in the 4 groups. As shown in **Figure 2A**, there was a downward trend in the Shannon index and Chao1 index from the NS to SCCS groups. **Figure 2C** shows that there were significant differences in the Bray-Curtis distance matrix at the genus level among groups among the NS ,LGDS, HGDS,SCCS groups (P=0.001). As shown in **Figures S1A–C**, there were significant differences between the NS and SCCS groups in the Shannon index and Chao1 index between the NS and LGDS groups (*P<* 0.05). With respect to β diversity, **Figures S2A–C** shows that there were significant differences in the Bray-Curtis distance matrix at the genus level between the NS and LGDS, NS and HGDS, NS and SCCS groups (P=0.001).

**Figure 3:**
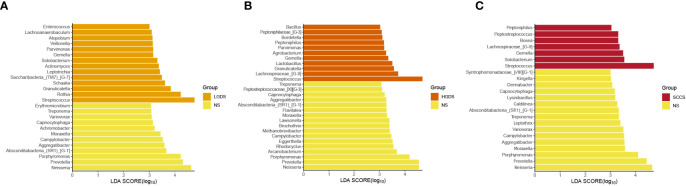
Linear discriminant analysis effect size (LEfSe) analysis of the abundance patterns of bacterial taxa of saliva samples. **(A)** The LEfSe analysis of the NS and LGDS. **(B)** The LEfSe analysis of the NS and HGDS. **(C)** The LEfSe analysis of the NS and SCCS.

**Figure 4:**
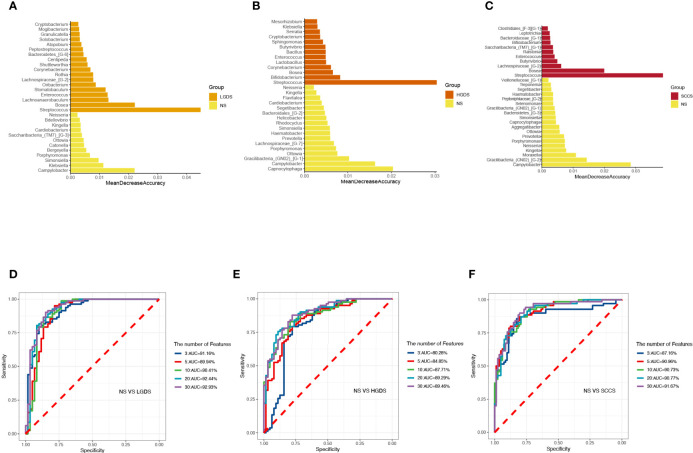
The randomForest function was used to perform random forest analysis. Extract the features in the random forest analysis results. Sort the features in descending order according to the MeanDecreaseAccuracy value. Take the top 3, 5, 10, 20, and 30 features in the ranking results to perform random forest analysis. Then, the pROC package was used to perform ROC analysis based on the random forest results, and ggroc was used to draw the result graph. According to the results of random forest and ROC, the species was selected with features of 30 to plot. The length of the bar graph was the value of MeanDecreaseAccuracy in the random forest analysis. Then, the abundance of the species corresponding to features in each sample group was counted. **(A)** The random forest model results tested by 30 genera of NS and LGDS. **(B)** The random forest model results tested by 30 genera of NS and HGDS. **(C)**, The random forest model results tested by 30 genera of NS and SCCS. **(D)** The top 3–30 genera were tested by ROC analysis of NS and LGDS. **(E)** The top 3–30 genera were tested by ROC analysis of NS and HGDS. **(F)** The top 3–30 genera were tested by ROC analysis of NS and SCCS.

The LEfSe results (**Figures 3A–C**) showed that at the genus level, the biomarkers for SCCS were Peptoniphilus, Peptostreptococcus, Bosea, Lachnospiraceae_[G-9], Gemella, Solobaterium, and Streptococcus. The biomarkers for HGDS were Bacillus, Peptoniphilaceae_[G-3], Bordetella, Peptoniphilus, Parvimonas, Agrobacterium, Gemella, Lactobacillus, Granulicatella, Lachnospiraceae_[G-9], and Streptococcus. The biomarkers for LGDS were Enterococcus, Lachnnoanaerobaculum, Atopobium, Veillonella, Parvimonas, Gemella, Solobaterium, Actinomyces, Leptotrichia, Saccharibacteria_(TM7)_[G-7], Schaalia, Granulicatella, Rothia, and Streptococcus. To determine whether the gut microbiota can be used as a biomarker to differentiate LGD, HGD, and SCC patients from healthy controls, we constructed 5 random forest models using all the microbiota components at the genus level. The genera for the random forest were selected according to the value of the species MeanDecreaseAccuracy in the analysis result of the varImpPlot function in descending order. **Figures 4A–C** show the random forest model results tested by 30 genera of NS and LGDS, NS and HGDS, NS and SCCS. As shown in **Figures 4D–F**, the top 5 genera in the LGDS, HGDS, and SCCS groups had predictive values with AUCs of 89.94%, 84.85%, and 90.96%, respectively.

Next, we compared the similarities and differences between the characteristic bacteria found in LEfSe and random forest. The common characteristic bacteria of every group were shown in **Table 2**.

**Table 2:** The common characteristic bacteria of the LGDS, HGDS, and SCCS groups found in LEfSe and random forest.

	LEfSe	Random forest
LGDS group	*Atopobium*	
	*Enterococcus*	
	*Granulicatella*	
	*Lachnnoanaerobaculum*	
	*Rothia*	
	*Solobaterium*	
	*Streptococcus*	
HGDS group	*Bacillus*	
	*Lactobacillus*	
	*Streptococcus*	
SCCS group	*Bosea*	
	*Streptococcus*	

**Figure 5:**
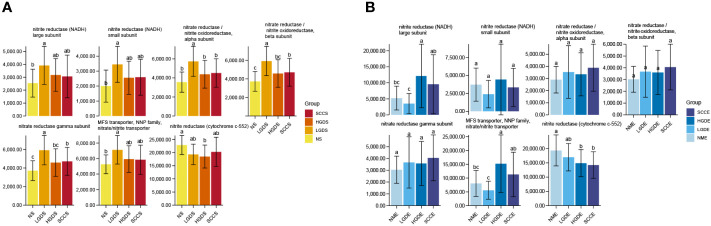
Changes in nitrate/nitrite reductase functions in the microbiota of saliva and brush samples. The LSD test function of the R software agricolae package was used to analyze the difference in the α diversity index of nitrite metabolism, and the fdr method was used to correct the P value. **(A)** Changes in nitrate/nitrite reductase functions in the microbiota of saliva samples. **(B)** Changes in nitrate/nitrite reductase functions in the microbiota of brush samples. Different small letters in the bar chart represent statistical differences between the two sets of data.

Accumulating evidence has reported that the microbiota might produce secondary metabolites, such as reactive nitrate and nitrite, which are carcinogens associated with cancer development. We next compared all groups regarding the microbial functional signatures involved in nitrate and nitrite reductase by PICRUSt2. The results predicted that there were significant differences between the NS and SCCS groups in nitrite oxidoreductase alpha subunit, nitrite oxidoreductase beta subunit, and nitrite reductase gamma subunit (**Figure 5A**).

### Microbial Differences Among Brush Specimens From Different Pathological Groups

The brush specimens from the different pathological groups (the normal group, LGD, HGD, and ESCC group) are indicated as NME, LGDE, HGDE and SCCE, respectively.

The results presented in **Figures 1C, D** show the compositions of the microbiota in the 4 groups. As shown in **Figure 2B**, there was a downward trend in the Shannon index and Chao1 index from the NME to SCCE groups. **Figure 2D** shows that there were significant differences in the Bray-Curtis distance matrix at the genus level among groups among the NME, LGDE, HGDE, SCCE groups (P=0.001). As shown in **Figures S1D–F**, there were significant differences between the NME and LGDE, NME and HGDE, NME and SCCE groups in the Shannon and Chao1 indices (*P<* 0.05). With respect to β diversity, **Figures S2D, E** shows that there were significant differences in the Bray-Curtis distance matrix at the genus level among groups between the NME and SCCE, NME and HGDE, NME and SCCE groups (P=0.001).

**Figure 6:**
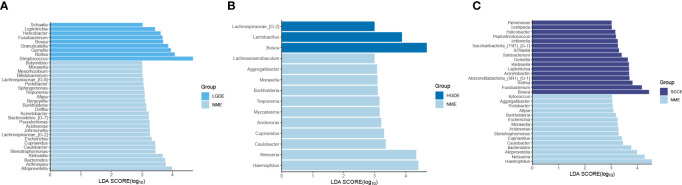
Linear discriminant analysis effect size (LEfSe) analysis of the abundance patterns of bacterial taxa of saliva samples. **(A)** The LEfSe analysis of the NME and LGDE. **(B)** The LEfSe analysis of the NME and HGDE. **(C)** The LEfSe analysis of the NME and SCCE.

**Figure 7:**
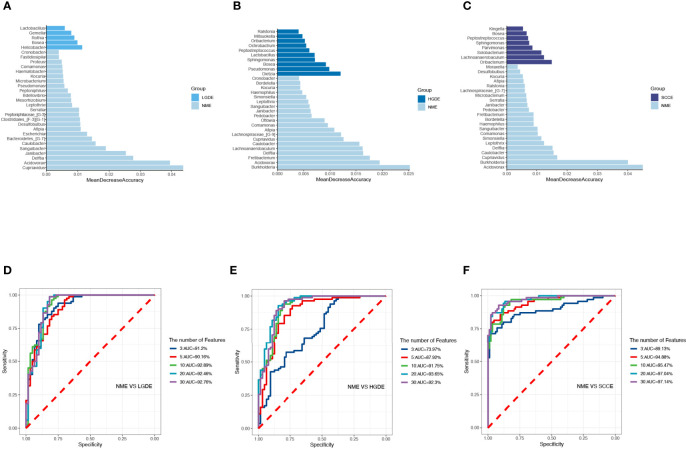
The randomForest function was used to perform random forest analysis. Extract the features in the random forest analysis results. Sort the features in descending order according to the MeanDecreaseAccuracy value. Take the top 3, 5, 10, 20, and 30 features in the ranking results to perform random forest analysis. Then, the pROC package was used to perform ROC analysis based on the random forest results, and ggroc was used to draw the result graph. According to the results of random forest and ROC, the species was selected with features of 30 to plot. The length of the bar graph was the value of MeanDecreaseAccuracy in the random forest analysis. Then, the abundance of the species corresponding to features in each sample group was counted. **(A)** The random forest model results tested by 30 genera of NME and LGDE. **(B)** The random forest model results tested by 30 genera of NME and HGDE. **(C)** The random forest model results tested by 30 genera of NME and SCCE. **(D)** The top 3–30 genera were tested by ROC analysis of NME and LGDE. **(E)** The top 3–30 genera were tested by ROC analysis of NME and HGDE. **(F)** The top 3–30 genera were tested by ROC analysis of NME and SCCE.

The LEfSe results (**Figures 6A-C**) showed that at the genus level, the biomarkers for SCCE were Parvimonas, Centipeda, Helicobacter, Peptostreptococcus, Saccharibacteria_(TM7)_[G-1], Schaalia, Solobacterium, Gemella, Kebsiella, Leptotrichia, Acinetobacter, Absconditabacteria_(SR1)_[G-1], Rothia, Fusobacterium, and Bosea. The biomarkers for HGDE were Lachnospiraceae_[G-1], Lactobacillus and Bosea. The biomarkers for LGDE were Schaalia, Leptotrichia, Helicobacter, Fusobacterium, Granulicatella, Gemella, Rothia, and Streptococcus. To determine whether the gut microbiota can be used as a biomarker to differentiate LGD, HGD, and SCC patients from healthy controls, we constructed 5 random forest models using all the microbiota components at the genus level. The genera for the random forest were selected according to the value of the species MeanDecreaseAccuracy in the analysis result of the varImpPlot function in descending order. **Figures 7A–C** show the random forest model results tested by 30 genera of NME and LGDE, NME and HGDE, NME and SCCE. As shown in **Figures 7D–F**, the top 5 genera in the LGDE, HGDE, and SCCE groups had predictive value with AUCs of 90.16%, 87.92%, and 94.88%, respectively.

Next, we compared the similarities and differences between the characteristic bacteria found in LEfSe and random forest. The common characteristic bacteria of every group were shown in **Table 3**.

**Table 3:** The common characteristic bacteria of the LGDE, HGDE, and SCCE groups found in the LEfSe and random forest.

	LEfSe	Random forest
LGDE group	*Bosea*	
	*Gemella*	
	*Helicobacter*	
	*Rothia*	
HGDE group	*Bosea*	
	*Lactobacillus*	
SCCE group	*Bosea*	
	*Parvimonas*	
	*Peptostreptococcus*	
	*Solobacterium*	

We next compared all groups regarding the microbial functional signatures involved in nitrate and nitrite reductase by PICRUSt2. The results predicted that nitrite reductase (cytochrome c-552) decreased significantly in the SCCE and HGDE groups compared with the NME group (**Figure 5B**).

### Microbiota Associations and Differences Between Paired Saliva and Lesion Samples From Patients in the Same Pathological Group

**Figure 8:**
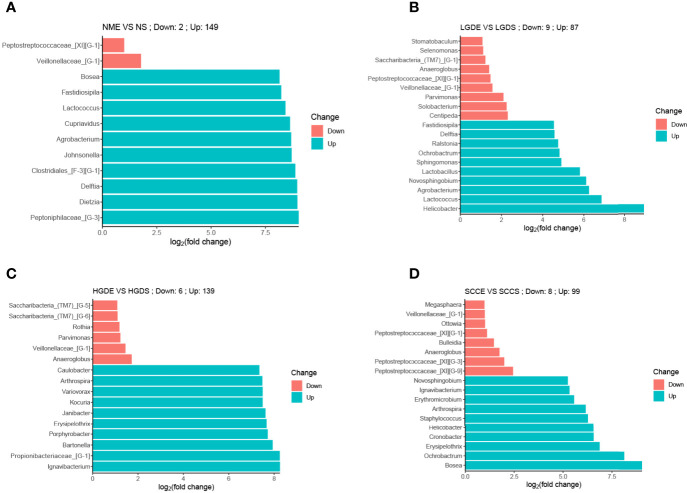
Bar charts of differences in relative abundances at the genus level between paired saliva and brush samples (analyzed by DESeq2). **(A)** Bar chart of differences in relative abundances at the genus level between paired saliva and brush samples in normal people. **(B)** Bar chart of differences in relative abundances at the genus level between paired saliva and brush samples in patients with LDG. **(C)** Bar chart of differences in relative abundances at the genus level between paired saliva and brush samples in patients with HDG. **(D)** Bar chart of differences in relative abundances at the genus level between paired saliva and brush samples in patients with SCC.

At the genus level, the bar chart shows the decrease and increase in relative abundance in paired samples. The data was calculated by DESeq2. We found that in all paired samples, the relative abundance of *Veillonellaceae_[G-1]* was decreased in the brush samples (**Figure 8**).

**Figure 9:**
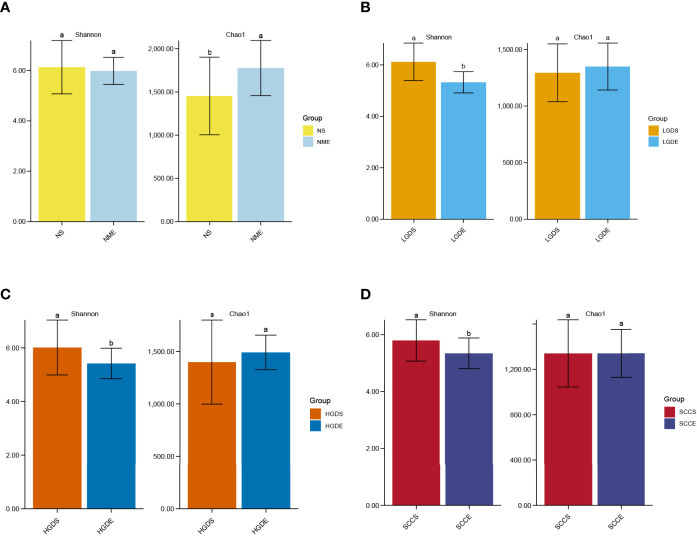
The α diversities at the genus level between paired saliva and brush samples. **(A)** The Shannon and Chao1 indices of the NS and NME groups. **(B)** The Shannon and Chao1 indices of the LGDS and LGDE groups. **(C)** The Shannon and Chao1 indices of the HGDS and HGDE groups. **(D)** The Shannon and Chao1 indices of the SCCS and SCCE groups. Different small letters in the bar chart represent statistical differences between the two sets of data.

As shown in **Figure 9A**, significant differences were observed in the Chao1 indices between the NS and NME groups (*P<* 0.05). In addition, the Shannon indices were greatly decreased in the LGDE (*P<* 0.05) (**Figure 9B**), HGDE (*P<* 0.05) (**Figure 9C**), and SCCE (*P<* 0.05) groups (**Figure 9D**).

## Discussion

The esophagus is a tube-shaped muscle structure approximately 20–27 cm long connecting the mouth and stomach. With the continuous development of molecular technology, 16S rRNA gene sequencing using second-generation sequencing techniques has gradually become widely used to research the human microbiota. These methods can be used to quantitatively describe not only the flora present in a complex biological matrix but also the entire community and its constituents (Fillon et al., 2012).

Few studies have examined the composition of the microbiota in ESCC lesions. Our results showed, for the first time, microbial changes in paired saliva and cell brush samples from healthy individuals and LGD, HGD, and ESCC patients. Previous studies have confirmed that BMI, smoking, and alcohol consumption are associated with changes in the esophageal microbiota (Okereke et al., 2019). Therefore, in our present study, relevant factors were controlled at the time of enrollment. We observed that as the disease progressed, the α diversity in the saliva and cell brush samples decreased, consistent with the findings of Chen et al., Yu et al. and Peters BA et al. (Yu et al., 2014; Chen et al., 2015; Peters et al., 2017; Yang et al., 2021). At the phylum level, in the saliva and cell brush specimens, the composition of the microbiome changed with disease progression, with the altered taxa being different in each group. At the genus level, in both the saliva and cell brush specimens, *Granulicatella, Rothia, Streptococcus*, *Gemella, Leptotrichia* and *Schaalia* were common biomarkers in patients with LGD, and *Lactobacillus* was a common biomarker in patients with HGD. *Bosea*, *Solobacterium, Gemella*, and *Peptostreptococcus* were common biomarkers in patients with esophageal cancer. Notably, the top 3 genera in the saliva and cell brush specimens had AUCs of 87.16 and 89.13%, respectively, which were higher than those reported by Li et al. (2020). This result suggests that regardless of the sample type, changes in microecological conditions have the ability to predict the occurrence of esophageal cancer. In a study by Liu et al. (2018), the abundances of *Streptococcus* and *Prevotella* were suggested to be associated with the advanced type of esophageal cancer. In our present study, the abundance of *Streptococcus* was increased significantly in the SCCS group, while that of *Prevotella* was significantly decreased. Yang et al. observed a significant overrepresentation of *Fusobacteria*, *Bacteroidetes*, and *Spirochaetes* (P < 0.001) and lower abundances of *Proteobacteria* and *Thermi* in ESCC tissue (Yang et al., 2021). In our present study, similar findings were observed for the brush samples. The relative abundance of *Fusobacteria* was greatly increased in ESCC patients. Similar results were also observed in Japanese patients with esophageal cancer (Yamamura et al., 2019).

In the study of Wang et al. (Wang et al., 2019), their LEfSe analysis found that in the saliva sample, the high risk of ESCC may be related to *Actinomyces* and *Atopobium*, while the healthy control group is closely related to *Fusobacterium* and *Porphyromonas* (the analysis was performed at the genus level). Our LEfSe analysis also pointed out that *Porphyromonas* was a biomarker for normal people.

In the present study, we also observed that the microbiota in the paired saliva and brush samples were different. The differences in the dominant bacteria observed between the NS and NME groups were similar to the findings of Dong et al. (2018). Importantly, this is the first study to show the differences in the dominant bacteria between the LGDS and LGDE, HGDS and HGDE, and SCCS and SCCE groups. Interestingly, at the phylum level, *Bacteroidota*, *Actinobacteriota*, and *Cyanobacteria* were significantly more abundant in the saliva samples from the disease groups than in the paired brush samples. The genus *Neisseria* was significantly more abundant in the saliva samples from the disease groups than in the paired brush samples. Peter et al. observed that depletion of the commensal genus *Neisseria* was associated with a lower esophageal adenocarcinoma (EAC) risk (Peters et al., 2017). Therefore, the role of *Neisseria* in the occurrence and development of esophageal cancer requires further study.

PICRUSt2 contains an updated, enlarged gene family and reference genome database that is interoperable with any operational taxonomic unit (OTU) screening, ASV or denoising algorithm and can make phenotype predictions. Benchmarks show that PICRUSt2 is generally more accurate than PICRUSt1 and other competing methods (Douglas et al., 2020). Using PICRUSt1, Yang (Yang et al., 2021) observed that some KEGG pathways were significantly enriched in ESCC tissue, including aminoacyl-tRNA biosynthesis, translation proteins, ribosome biogenesis, ribosomes, etc. and ESCC microbiota had altered nitrate reductase functions compared with the normal group. Our results identified similar results in brush samples in which patients with ESCC had decreased nitrate reductase functions. However, the salvia samples had different results, which need further study.

There are several limitations of the present study. First, all participants in this study were from a population in northern China. Because there are many factors that affect the esophageal microbiota, the relevant conclusions made in the present study may be slightly different from those of other studies. Second, although this study used the largest esophageal cancer, precancerous lesion, and normal patient sample sizes among all previously published microbiota research articles, the datasets in each group were still small compared to those analyzed for other diseases.

## Conclusion

The microbiota in saliva and on the surface of esophageal cancer lesions changed with esophageal cancer progression, and the microbiota in saliva and esophageal cell brush samples were not the same. In summary, our results showed that ESCC-specific microbial groups may serve as sensitive and specific clinical diagnostic markers. It is possible that targeting these bacterial strains may be effective and beneficial in diagnosing ESCC, and accurate microbiological assessments will aid in future ESCC detection and treatment.

## Data Availability Statement

The original contributions presented in the study are included in the article/**Supplementary Material**. Further inquiries can be directed to the corresponding authors.

## Ethics Statement

The studies involving human participants were reviewed and approved by Ethics Committee of the National Cancer Center/Cancer Hospital, Chinese Medical College and Peking Union Medical College. The patients/participants provided their written informed consent to participate in this study.

## Author Contributions

GW, WZ, SH, YZ, ZL, LD, and YL drafted the protocol and collected specimens at the Cancer Hospital of the Chinese Academy of Medical Sciences (Beijing, China). DZ, CH, and GS assisted ZL in collecting specimens at Feicheng, Linzhou, and Cixian. ZL performed the statistical analysis. All authors contributed to the article and approved the submitted version.

## Funding

This research was supported by grants from (1) the National Key Research and Development Program of China (grant no. 2016YFC1302800, 2016YFC0901402, 2018YFC1313103); (2) the Beijing Science and Technology Project (D17110002617002); (3) the CAMS Innovation Fund for Medical Sciences (CIFMS), (grant no. 2016-I2 M-001, 2017-I2 M-1–001, 2019- I2 M-2–004);(4) the Sanming Project of Medicine in Shenzhen (No. SZSM2019110080); (5) the PUMC Youth Fund and the Fundamental Research Funds for the Central Universities (grant no. 2017320012); and (6) the PUMC Graduate Innovation Fund (grant No. 2019–1002–81).

## Conflict of Interest

The authors declare that the research was conducted in the absence of any commercial or financial relationships that could be construed as a potential conflict of interest.

## Publisher’s Note

All claims expressed in this article are solely those of the authors and do not necessarily represent those of their affiliated organizations, or those of the publisher, the editors and the reviewers. Any product that may be evaluated in this article, or claim that may be made by its manufacturer, is not guaranteed or endorsed by the publisher.

## Supplementary Material

Supplementary Figure 1 The α diversities of microbiota in all groups. The LSD test function of the R software agricolae package was used to analyze the difference in the α diversity index of ASV, and the fdr method was used to correct the P value (P=0.05). **(A)** The Shannon and Chao1 indices of the NS and LGDS groups. **(B)** The Shannon and Chao1 indices of the NS and HGDS groups. **(C)** The Shannon and Chao1 indices of the NS and SCCS groups. **(D)** The Shannon and Chao1 indices of the NME and LGDE groups. **(E)** The Shannon and Chao1 indices of the NME and HGDE groups. **(F)** The Shannon and Chao1 indices of the NME and SCCE groups.

Supplementary Figure 2 The β diversities of microbiota in all groups. The vegdist vegan package was used to calculate the Bray-Curtis distance matrix at the genus level. Then, the cmdscale function was used for principal coordinate analysis, and the perMANOVA test was conducted by the adonis function. **(A)** The Bray-Curtis distance matrix at the genus level for the NS and LGDS groups. **(B)** The Bray-Curtis distance matrix at the genus level for the NS and HGDS groups. **(C)** The Bray-Curtis distance matrix at the genus level for the NS and SCCS groups. **(D)** The Bray-Curtis distance matrix at the genus level for the NME and LGDE groups. **(E)** The Bray-Curtis distance matrix at the genus level for the NME and HGDE groups. **(F)** The Bray-Curtis distance matrix at the genus level for the NME and SCCE groups.
