# Supplementary figures and images for: Characterization of the Oral and Esophageal Microbiota in Esophageal Precancerous Lesions and Squamous Cell Carcinoma
Source: Front Cell Infect Microbiol. 2021 Sep 15;11:714162. doi: 10.3389/fcimb.2021.714162 (PMC8479167; doi:10.3389/fcimb.2021.714162)

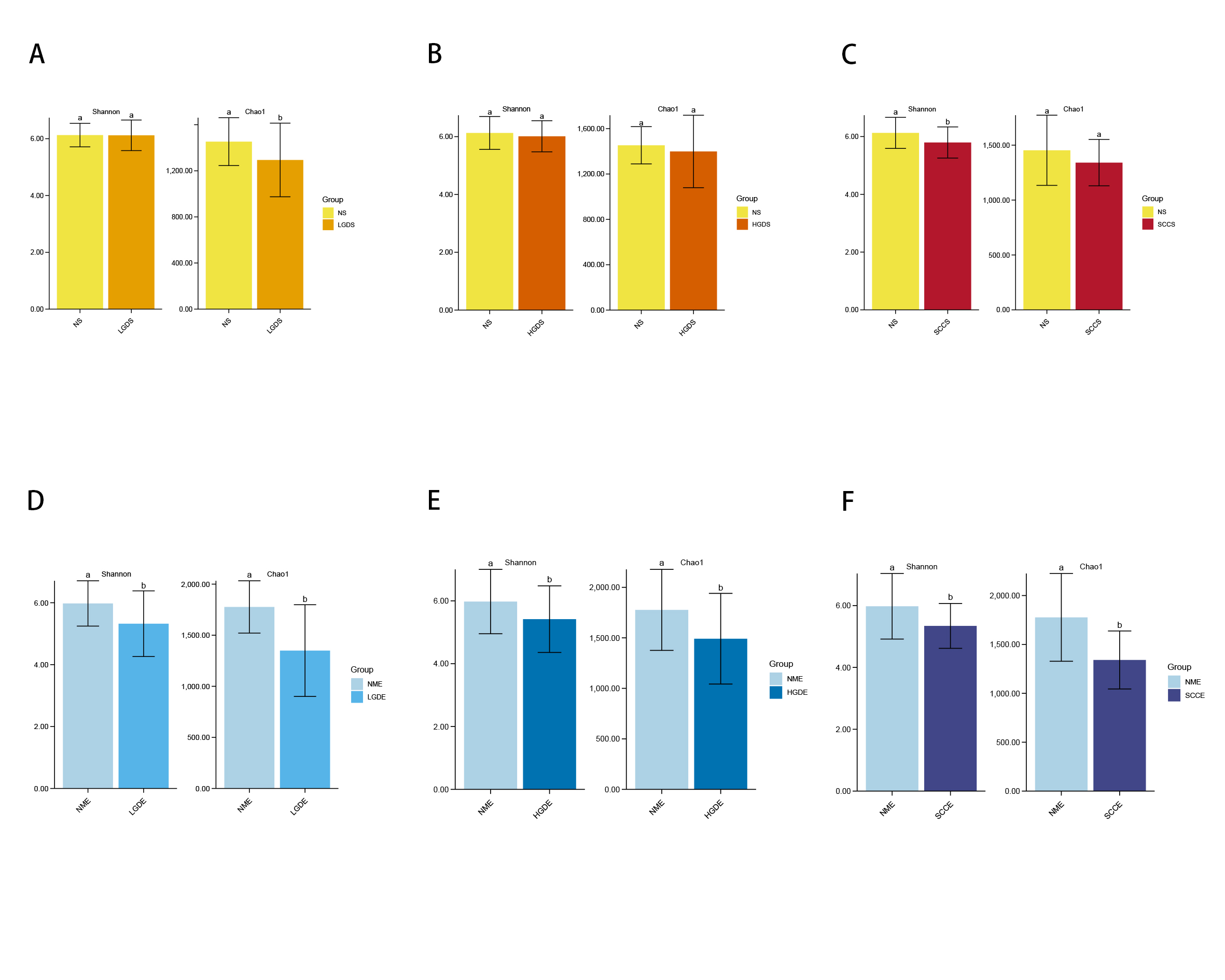

Supplement: Supplementary Figure 1 — The α diversities of microbiota in all groups. The LSD test function of the R software agricolae package was used to analyze the difference in the α diversity index of ASV, and the fdr method was used to correct the P value (P=0.05). (A) The Shannon and Chao1 indices of the NS and LGDS groups. (B) The Shannon and Chao1 indices of the NS and HGDS groups. (C) The Shannon and Chao1 indices of the NS and SCCS groups. (D) The Shannon and Chao1 indices of the NME and LGDE groups. (E) The Shannon and Chao1 indices of the NME and HGDE groups. (F) The Shannon and Chao1 indices of the NME and SCCE groups. [file Image_1.jpeg]

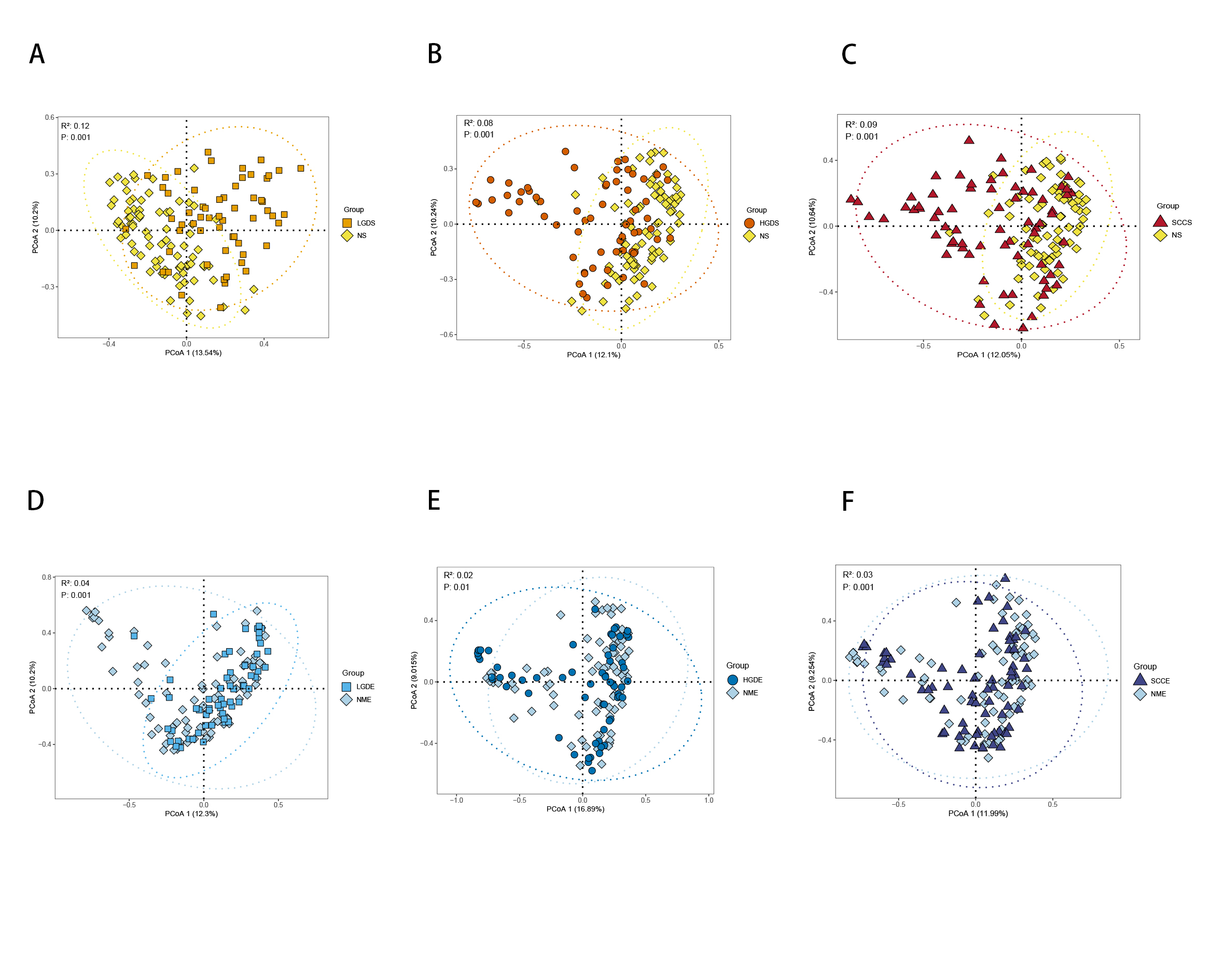

Supplement: Supplementary Figure 2 — The β diversities of microbiota in all groups. The vegdist vegan package was used to calculate the Bray-Curtis distance matrix at the genus level. Then, the cmdscale function was used for principal coordinate analysis, and the perMANOVA test was conducted by the adonis function. (A) The Bray-Curtis distance matrix at the genus level for the NS and LGDS groups. (B) The Bray-Curtis distance matrix at the genus level for the NS and HGDS groups. (C) The Bray-Curtis distance matrix at the genus level for the NS and SCCS groups. (D) The Bray-Curtis distance matrix at the genus level for the NME and LGDE groups. (E) The Bray-Curtis distance matrix at the genus level for the NME and HGDE groups. (F) The Bray-Curtis distance matrix at the genus level for the NME and SCCE groups. [file Image_2.jpeg]
